# Methadone Maintenance Treatment Reduces the Vulnerability of Drug Users on HIV/AIDS in Vietnamese Remote Settings: Assessing the Changes in HIV Knowledge, Perceived Risk, and Testing Uptake after a 12-Month Follow-Up

**DOI:** 10.3390/ijerph15112567

**Published:** 2018-11-16

**Authors:** Tam Minh Thi Nguyen, Bach Xuan Tran, Mercerdes Fleming, Manh Duc Pham, Long Thanh Nguyen, Huong Thi Le, Anh Lan Thi Nguyen, Huong Thi Le, Thang Huu Nguyen, Van Hai Hoang, Xuan Thanh Thi Le, Quan Hoang Vuong, Manh Tung Ho, Van Nhue Dam, Thu Trang Vuong, Ha Ngoc Do, Vu Nguyen, Huong Lan Thi Nguyen, Huyen Phuc Do, Phuong Linh Doan, Hai Hong Nguyen, Carl A. Latkin, Cyrus SH Ho, Roger CM Ho

**Affiliations:** 1 Vietnam Authority of HIV/AIDS Control, Ministry of Health, Hanoi 100000, Vietnam; minhtam71.moh@gmail.com (T.M.T.N.); manh65vn@yahoo.com (M.D.P.); nguyenlan97@yahoo.com (L.T.N.); lehuongvaac@gmail.com (H.T.L.); haihrp@yahoo.com (H.H.N.); 2 Institute for Preventive Medicine and Public Health, Hanoi Medical University, Hanoi 100000, Vietnam; lethihuong@hmu.edu.vn (H.T.L.); thangtcyt@gmail.com (T.H.N.); hoanghaivan2002@yahoo.com (V.H.H.); lethithanhxuan@hmu.edu.vn (X.T.T.L.); 3 Bloomberg School of Public Health, Johns Hopkins University, Baltimore, MD 21205, USA; carl.latkin@jhu.edu; 4 School of Medicine and Medical Science, University College Dublin, Belfield, D04 V1W8 Dublin, Ireland; mercyfleming@gmail.com; 5 National Institute of Hygiene and Epidemiology, Hanoi 100000, Vietnam; lananh_2003@yahoo.com; 6 Center for Interdisciplinary Social Research, Thanh Tay University, Hanoi 100000, Vietnam; hoang.vuong@thanhtay.edu.vn; 7 Solvay Brussels School of Economics and Management, Centre Emile Bernheim, Université Libre de Bruxelles, B-1050 Brussels, Belgium; 8 Institute of Philosophy, Vietnam Academy of Social Sciences, Hanoi 100000, Vietnam; tung.ho@wu.edu.vn; 9 Faculty of Graduate Studies, National Economics University, Hanoi 100000, Vietnam; sshpa.2017@gmail.com; 10 Sciences Po Paris, Campus de Dijon, 21000 Dijon, France; thutrang.vuong@sciencespo.fr; 11 Youth Research Institute, Ho Chi Minh Communist Youth Union, Hanoi 100000, Vietnam; ngochayri@gmail.com; 12 Department of Neurosurgery Spine-Surgery, Hanoi Medical University Hospital, Hanoi 100000, Vietnam; nguyenvu@hmu.edu.vn; 13 Institute for Global Health Innovations, Duy Tan University, Danang 550000, Vietnam; huong.ighi@gmail.com; 14 Center of Excellence in Evidence-Based Medicine, Nguyen Tat Thanh University, Ho Chi Minh City 700000, Vietnam; huyen.ighi@gmail.com (H.P.D.); linh.ighi@gmail.com (P.L.D.); 15 Department of Psychological Medicine, National University Hospital, Singapore 119074, Singapore; cyrushosh@gmail.com; 16 Department of Psychological Medicine, Yong Loo Lin School of Medicine, National University of Singapore, Singapore 119228, Singapore; hocmroger@yahoo.com.sg; 17 Center of Excellence in Behavioral Medicine, Nguyen Tat Thanh University, Ho Chi Minh 700000, Vietnam

**Keywords:** knowledge, attitudes and practices, Vietnam, methadone maintenance, HIV/AIDS

## Abstract

Methadone Maintenance Treatment (MMT) program has been considered a medium through which human immunodeficiency virus (HIV) risks assessment and prevention on drug use/HIV-infected population can be effectively conducted. Studies concerning the implementation of such idea on patients in remote, under-developed areas, however, have been limited. Having the clinics established in three mountainous provinces of Vietnam, this study aimed to evaluate the changes in knowledge of HIV, perceived risk, and HIV testing uptake of the patients. A longitudinal study was conducted at six MMT clinics in three provinces with a pre- and post-assessments among 300 patients. Outcomes of interest were compared between baseline and after 12 months. The magnitude of changes was extrapolated. The proportion of participants reporting that their HIV knowledge was not good fell by 4.4% (61.3% at the baseline vs. 56.8% at 12 months). The significant improvement seen was in the knowledge that needle sharing was a mode of transmission (82.7% vs. 89.6%). Nevertheless, the majority of participants reportedly considered mosquitoes/insect and eating with the HIV-infected patient were the route of transmission at both time points (84.7% vs. 89.1%, 92.2% vs. 93.3%, respectively). This study found a limited improvement in HIV knowledge and testing uptake among MMT patients following a 12-month period. It also highlighted some shortcomings in the knowledge, attitudes and practices (KAP) of these patients, in particular, incorrect identification of HIV transmission routes, among patients both at program initiation and follow-up. The findings lent support to the argument for enhancing education and counseling efforts at MMT clinics regarding HIV, as well as for improving access to preventive and health care services through the integration of MMT/HIV services.

## 1. Introduction

Methadone maintenance treatment (MMT) is seen globally as one of the most effective methods, both in terms of cost and patient outcome, to help people withdraw from using opioid drugs [1,2]. Many countries have introduced programs to assist in reducing the number of people using these drugs [3] so as to prevent morbidity and mortality directly in relation to opioid drugs, but also due to co-morbidities, such as infection [4,5]. MMT has shown efficacy in improving the quality of life of drug users and reducing their need to use other drugs in several studies worldwide, such as in China [6,7] and the US [8,9]. In the US in 2007, intravenous drug use was estimated to cause an economic burden of $193 billion, mainly due to loss of productivity and absenteeism [10]. Reducing this economic burden of drug use through methadone maintenance is seen as highly advantageous, in and can lead to greater numbers of drug users in the workforce and therefore greater productivity [11].

In Vietnam, there are roughly 260,000 HIV patients [12] and 180,000 people who inject drugs (PWID) [13]. Both groups are strongly interlinked as around 30% of the HIV positive population are PWID [13,14]. HIV in Vietnam is largely concentrated among the drug using population so targeting of this group through MMT services to reduce HIV is of benefit. Services for both have been rapidly increased in recent years to now include 364 HIV outpatient centers [15] and 251 MMT centers [16]. This improvement has been mainly fueled by investment from international AID organizations, such as PEPFAR and the Global AIDS fund; however, more recently, funding has declined. Maximizing the public benefit from both services is therefore very important. Using MMT services to target HIV patients or those at risk of HIV has already been studied in Vietnam. While drug use and HIV transmission have already been linked [17], alterations to drug usage through MMT programs have been shown to improve HIV/AIDS patient quality of life within the country [18]. MMT services could, therefore, be used to assess HIV risks and prevention among this vulnerable group of patients.

Currently, most data in Vietnam regarding MMT services in relation to HIV knowledge, attitudes and practices (KAP) focuses mainly on urban areas and cities [19]. There is substantially less information regarding MMT and HIV in mountainous provinces. People here have been identified as having increased rates of health problems, including drug use and HIV remain among others [20]. Access to services in these areas is also limited giving rise to a larger hidden drug using population (those who have not presented to medical professionals as drug users) and more people who do not know their HIV status [21]. As more patients utilize MMT services in these regions, the hope is that information regarding HIV will also become more prevalent. To maximize health services resources, examining the effectiveness of MMT programs in improving the KAP of HIV/AIDS is important. Ideally, as patients utilize the services, they should improve their KAP towards HIV and take this into the community to reduce the transmission and risk of HIV/AIDS among the hidden drug using population as well. This study aims to evaluate the changes in HIV knowledge, perceived risk and HIV testing uptake among MMT patients follow a year of treatment. This could help to identify areas where improvements are needed.

## 2. Materials and Methods

### 2.1. Study Setting and Population

Pre- and post-assessments with no control group from December 2014 to December 2015 in six clinics in three mountainous provinces namely Dien Bien, Lai Chau, and Yen Bai province. Six clinics were selected, including Provincial AIDS Centers in Dien Bien, Lai Chau, and Yen Bai City; and District Health Centers in Thanh Xuong, Phong Tho and Nghia Lo. We invited patients who met following inclusion criteria: (1) Initiating MMT service at one of the selected clinics at the time of the baseline assessment in 2014; (2) volunteering and agreeing to participate in research by giving written consent; and (3) having good physical and mental health to be able to answer the questionnaire. MMT patients were interviewed in the first month and 12th-month of MMT program.

In this study, the sample size was calculated using the formula for estimating the difference between two population means. With absolute precision ∆ = 0.05 and standardized mean difference = 0.18 [22], the sample size required in each province was 100. The total sample size was 300 patients. The study first screened 300 patients participating in the MMT program; after 12 months of treatment, 56 patients withdrew, and 244 patients remained. We compared results in the first month and 12th-month of 244 remaining patients. The percentage of male participants in our sample was 100%, since only male patients registered at the clinics we studied at the time of investigation.

### 2.2. Measures and Instruments

Face-to-face interviews were conducted by well-trained researchers at the Hanoi Medical University using a structured questionnaire in Vietnamese. The questionnaire was piloted in the small group with 10 patients. After receiving feedback from patients regarding logical, language and text perspectives, we revised the questionnaire to make sure that the content was appropriate to the context of study, as well as the quality of data. Socio-demographic characteristics were collected consisting of age, gender, ethnicity, education, marital status, and occupation.

#### 2.2.1. Knowledge of HIV Treatment and Prevention

The knowledge included: (1) HIV/AIDS transmission; (2) susceptibility to HIV infection; and (3) HIV prevention according to five questions set by the Vietnam Ministry of Health (MOH) in 2007. The respondents were asked to answer five True/False questions. According to the guideline of the MOH, MMT patients had good knowledge if they correctly answered all of five questions, while others did not have good knowledge if any one of five questions was answered incorrectly.

#### 2.2.2. Attitudes of HIV Treatment and Prevention

The attitudes included: (1) Self-assessed HIV status; (2) reasons for risk of HIV/AIDS infection; and (3) reasons for no risk of HIV/AIDS infection.

#### 2.2.3. Practices of HIV Prevention and Treatment

The practices included: (1) Using HIV Testing and Counselling Services before beginning MMT; (2) provider-initiated testing and counseling; (3) HIV Testing; and (4) HIV Testing result.

### 2.3. Statistical Analysis

We described frequency (n) and prevalence (%) of variables at the baseline and in follow-up data. Chi-squared test and Fisher’s exact test were used to assess the difference between before and after MMT intervention. Statistical significance was set at *p*-value < 0.05. The effect size was performed to determine the effect of MMT program. Among effect size, Phi was used to estimates the extent of the relationship between two binary variables (2 × 2) and Cramér’s V was used with variables having more than two levels. In the context of effect size level classification, very small, small, medium and large effect sizes equating to the values 0.01, 0.2, 0.5 and 0.8, respectively were used [23]. Data analysis was performed by using STATA software version 12.0.

### 2.4. Ethics Approval

This study was approved by the Vietnam Authority of HIV/AIDS Control’s Scientific Research Committee.

## 3. Results

Among 244 patients in this study, majority belonged to the Kinh ethnic background (56.8%), had less than high school education (56.8%), were living with spouses or partners (57.5%) and being employed as freelancers (79.7%).

Table 1 depicts the change in knowledge of HIV/AIDS prevention and treatment among MMT patients over 12 months. Most participants thought a healthy-looking person cannot be HIV infected, both at the start of the study (86.4%) and after 12 months (87.9%). Significantly more participants at 12 months (89.6%82.7% at baseline) knew that sharing needles and syringes was a method of HIV transmission, the effect size of change for this indicator was 0.022. There were 94.7% and 96.7%, respectively, of participants at baseline and 12 month time point identified injecting drug users as being susceptible to HIV. Majority of participants at both time points identified faithfulness (88.0% at baseline89.6%) and condom use (97.9% at baseline92.9%) as being protective against HIV, however, they also incorrectly identified mosquitoes (84.7% at baseline89.1%) and sharing food (92.2% at baseline93.3%) as modes of transmission. Fewer participants at 12 months said their knowledge was not good (56.8%61.3% at baseline).

The changes to attitudes of HIV risk among MMT patients at 12 months are described in Table 2. Most patients at both times point perceived that they were not at risk of HIV (54.3% and 54.6%). Drug injecting was the most common reason identified for increased risk of HIV (75% and 72.1%) while 15.2% and 17.4% at 12 months also identified sex without using condoms as a reason for risk. 72.7% and 70.7% at 12 months identified not sharing needles as the main reason for no risk of HIV. Being faithful, using condoms and not having sex with female sex workers were other common reasons for no risk.

Table 3 depicts the changes to practices of MMT patients for HIV treatment and prevention. Slightly more patients had used HIV testing and counseling services at 12 months than at the start (86.8%81.4%). District level health bureaus were used by 91.8% of patients at the start and 94.1% of patients at 12 months for provider-initiated testing and counseling.

## 4. Discussion

In this study, we aimed to assess the level of the knowledge, attitudes, and practices (KAP) regarding HIV/AIDS of MMT patients in mountainous areas and possible improvement after one year of following MMT program. In general, the majority of our participants displayed fairly sufficient knowledge regarding HIV transmission and prevention, appropriate HIV risk perception and adequate HIV testing update. However, very limited changes were found regarding the KAP at 12 month follow up compared to program initiation. This highlighted the difficulties of enhancing HIV/AIDS KAP in less developed areas and called for greater educational and counseling efforts.

Over the course of 12 months, the only significant KAP improvement in our study was seen in the knowledge of MMT patients about sharing needles as HIV transmission mode, with a small effect size of the change. Such finding was in line with a study in China examining the long-term effects of methadone maintenance treatment that also demonstrated improvements in knowledge with regards to needles sharing [24]. Another study in Nepal concerning safer drug use interventions, such as sterile injecting equipment and education, also showed that knowledge of HIV of participants increased while unsafe practices decreased as they progressed with the intervention program [25]. Nonetheless, although MMT services have been expected to be a potentially beneficial route for targeting HIV risk and prevention as HIV prevalence among PWID was substantial [26], literature looking specifically into how MMT impacts the KAP of HIV have been limited. The lack of improvement in KAP found in our study suggested that there were probably insufficient educational efforts at MMT clinics to enhance HIV-related knowledge of MMT patients. To our knowledge, in mountainous MMT clinics, patients gained information mostly through health staffs-provided informal advice and routine counselling when coming for their daily MMT dose. Organized, formal educational campaigns on HIV-related issues were scarce.

In addition, while most participants correctly identified main HIV modes of transmission and methods of protection—transmission via needles sharing, protected by protected sex etc., a substantial proportion of respondents reportedly considering mosquitoes and food sharing as HIV transmission route. This suggests potentially serious shortcomings in HIV knowledge of those in mountainous areas, which could also be a result of the shortage of educational and counseling activities/programs available that otherwise should be included in MMT clinics. While MMT services may have been adequate at providing the actual methadone treatment, they seem to lack a holistic service and complete healthcare intervention for the patients. As the HIV epidemic in Vietnam is concentrated among PWID, to improve HIV testing uptake among PWID and to maximize the expenditure on services, it would be beneficial for the patients to have access to both MMT and HIV-related services simultaneously. MMT patients have also indicated their desire for integrated and decentralized services [27] and one study has demonstrated that when MMT services are integrated with HIV centers, HIV testing and counseling rates increase [28]. Such integrated approach to tackle HIV-related issues in drug-using patients would be of even greater help for a mountainous population in Vietnam, as previous studies have shown that geographical barriers, such as distance to services, which likely to undermine adherence to MMT program, would also hinder HIV service uptake [29].

Several implications can be drawn from this study. Greater efforts should be spent on improving the knowledge of MMT patients with regards to HIV risks, probably through educational and counseling programs provided by the staffs of the MMT clinics. Potentially more beneficial is the integration of MMT and HIV services within an MMT clinic, so that an MMT clinic would offer a comprehensive package of services covering MMT, HIV testing and HIV counseling. In the Vietnam context, the benefits of such comprehensive services-offering clinics have been appraised by different parties involving in these services, including clinic directors, providers, staffs, and patients [30]. Policy and clinical-level movements are recommended to be conducted, including issuing legal framework and policies facilitating integration, staff training and improving collaboration across different services providers to tackle documented barriers to such integration [30]. In addition, our findings suggested some directions for further researches on the subject of HIV-related KAP—in particular the sources from which patients obtain HIV-related information, the basis of their beliefs with regard to this knowledge, or whether there were any other influences during the time between points of data collection.

## 5. Limitations

Of 300 patients, only 244 participants completed the survey as 56 withdrew before the 12 month period was over. The withdrawal of patients, typically those with lower adherence to MMT and worse KAP toward HIV risks and prevention, may lead to bias in the changes seen. The patients that completed the study and adhered to treatments may already have better KAP about HIV risks and prevention even in absence of MMT enrolment, implying that there would be greater positive changes if the 56 patients were included. Although the sample was quite small, six centers across provincial and district level services were chosen hopefully making the data representative of MMT users generally in mountainous areas. There is a risk that the sample is slightly skewed towards people who know more about HIV than the generic drug using population as these patients are actively seeking medical care and therefore may care more about their health than the remaining hidden drug using population.

## 6. Conclusions

In conclusion, this study found a limited improvement in HIV knowledge and testing uptake among MMT patients following a 12-month period. It also highlighted some shortcomings in the KAP of these patients, in particular, incorrect identification of HIV transmission routes, among patients both at program initiation and follow-up. The findings lent support to the argument for enhancing education and counseling efforts at MMT clinics regarding HIV, as well as for improving access to preventive and health care services through the integration of MMT/HIV services.

## Figures and Tables

**Table 1:** Change in knowledge of HIV prevention and treatment among methadone maintenance treatment patients.

Characteristics	Baseline		12 Month Follow-Up		*p*-Value	Effect Size
	n	%	n	%		
**A healthy-looking person can be HIV-infected**						
No	210	86.4	211	87.9	0.62	0.022
Yes	33	13.6	29	12.1		
**HIV/AIDS transmission route**						
Blood Transfusion Unsafety	170	70.0	174	72.2	0.59	0.025
Sharing needles and syringes	201	82.7	216	89.6	0.03	0.100
Pass from mother to child	117	48.2	123	51.0	0.53	0.029
Unprotected sex	217	89.3	224	93.0	0.16	0.064
**Susceptibility to HIV infection**						
Injecting drug users	230	94.7	231	96.7	0.28	0.049
Sex workers	187	77.3	183	76.6	0.86	0.008
Long distance highway drivers	13	5.4	21	8.8	0.14	0.067
Multiple sex partners	78	32.2	98	41.0	0.05	0.091
**Faithfulness to partners: A means to prevent HIV infection**						
False	29	12.0	25	10.4	0.57	0.026
True	212	88.0	215	89.6		
**The correct use of the condom reduces the risk of HIV infection**						
False	5	2.1	17	7.1	0.01	0.122
True	238	97.9	221	92.9		
**A healthy or healthy-looking person can be HIV-positive**						
False	49	20.3	66	27.7	0.06	0.088
True	193	79.8	172	72.3		
**Mosquitoes/insect can transmit HIV**						
False	37	15.3	26	10.9	0.16	0.065
True	205	84.7	212	89.1		
**HIV infection is transmitted through eating with a person with HIV-infected**						
False	19	7.9	16	6.7	0.62	0.023
True	223	92.2	224	93.3		
**Knowledge about HIV/AIDS**						
Not good	93	38.8	102	43.2	0.32	0.045
Good	147	61.3	134	56.8		

**Table 2:** Change in perceived HIV risk among methadone maintenance treatment patients.

Characteristics	Baseline		Follow-Up		*p*-Value	Effect Size
	n	%	n	%		
**Self-assessed HIV status**						
No risk	121	54.3	114	54.6	0.94	0.018
Low risk	50	22.4	49	23.4		
High risk	52	23.3	46	22.0		
**Reasons for risk of HIV/AIDS infection**						
Many partners	17	17.2	11	12.8	0.41	0.061
Sex without using the condom	15	15.2	15	17.4	0.67	0.031
Drug injection	75	75.0	62	72.1	0.65	0.033
Receiving blood transfusions	6	6.1	7	8.1	0.58	0.041
**Reasons for no risk of HIV/AIDS infection**						
Being faithful	58	40.6	70	50.0	0.11	0.095
Using condom	32	22.4	37	26.4	0.43	0.047
Not sharing needles	104	72.7	99	70.7	0.71	0.022
Not having friends with HIV/AIDS infection	10	7.0	12	8.6	0.62	0.029
Not having anal sex	16	11.2	16	11.4	0.95	0.004
Not having sex with female sex workers	46	32.2	44	31.4	0.89	0.008
Not receiving blood transfusions	6	4.2	11	7.9	0.20	0.077

**Table 3:** Change in the uptake of HIV testing services among methadone maintenance treatment patients.

Characteristics	Baseline		Follow-Up		*p*-Value	Effect Size
	n	%	n	%		
**Using HIV Testing and Counselling Services**						
No	45	18.6	31	13.3	0.11	0.073
Yes	197	81.4	203	86.8		
**Provider-initiated testing and counseling**						
Commune Health Stations	20	8.2	14	5.9	0.31	0.046
District health bureau	223	91.8	225	94.1		
**HIV Testing result**						
Positive	38	17.1	46	20.3	0.14	0.094
Negative	170	76.6	175	77.1		
N/A	14	6.3	6	2.6		
